# Analysis of prognostic factors for pancreatic head cancer according to para‐aortic lymph node

**DOI:** 10.1002/cam4.853

**Published:** 2016-08-19

**Authors:** Jian‐yu Lin, Xing‐mao Zhang, Jian‐tiao Kou, Hua Fa, Xin‐xue Zhang, Yang Dai, Qiang He

**Affiliations:** ^1^ Comprehensive DepartmentBeijing Chaoyang HospitalCapital Medical UniversityBeijingChina; ^2^ Department of Hepatobiliary SurgeryBeijing Chaoyang HospitalCapital Medical UniversityBeijingChina

**Keywords:** Pancreatic cancer, para‐aortic lymph node, prognosis

## Abstract

This study was designed to investigate the relationship between prognosis of pancreatic head cancer and status of para‐aortic lymph node (PALN). A total of 233 patients with pancreatic head cancer who underwent surgical resection between February 2008 and October 2015 were enrolled in this study. Univariate and multivariate analyses were used to reveal the prognostic factors. Prognostic factors for patients with and without metastasis of PALN were analyzed, respectively. The 5‐year overall survival (OS) rate was 19.0% for all patients, and the positive rate of PALN metastasis was 18.9% (44/233). The 1‐, 2‐, 3‐, and 5‐year OS rates in patients without metastasis of PALN were 79.4%, 54.8%, 36.4%, and 22.9%, respectively, whereas the 1‐, 2‐, and 3‐year survival rates were 54.0%, 14.8%, and 0%, respectively, in patients with metastasis of PALN. Preoperative CA19‐9 level, tumor size, T status, N status, and adjuvant therapy were independent prognostic factors for all patients confirmed by multivariate analysis. For patients without PALN metastasis, back pain, tumor size, T status, N status, portal or superior mesenteric vein invasion, and adjuvant therapy were independent prognostic factors, while the only one influence factor for 2‐year OS was adjuvant therapy for patients with metastasis of PALN. Metastasis of PALN was associated with poor prognosis for patients with pancreatic head cancer. Patients with and without metastasis of PALN had different prognostic factors, and adjuvant therapy was the only prognostic factor for patients with metastasis of PALN.

## Introduction

Pancreatic cancer is one of the leading causes of cancer‐related death worldwide 1. Radical resection is still the only method for cure despite the improvement of adjuvant therapy. Unlike some other diseases such as colorectal cancer, gastric cancer, breast cancer, etc., the prognosis of pancreatic cancer has not improved during the last 20 years 2. The 5‐year overall survival (OS) rate of pancreatic cancer is reported to be only 6.8–25% after radical resection 3, 4, 5, 6. Less than 20% of patients with pancreatic cancer have the chance to receive operation due to the locoregional spread or distant metastasis at the time of diagnosis 7, 8, 9. As reported previously, prognosis of pancreatic cancer after operation is influenced by several factors including tumor size 10, 11, surgical margin status 12, 13, lymph node status 14, depth of invasion 15, and adjuvant therapy 3, 16. Para‐aortic lymph node (PALN) is regarded as extraregional lymph node, the necessity of removal of PALN for pancreatic head cancer remains unclear. Several studies show that metastasis of PALN is a sure sign of poor prognosis, and surgical resection should not be considered for pancreatic head cancer with definite metastasis of PALN 17, 18. In some other studies, however, patients can still benefit from surgical resection although metastasis of PALN 19. Thus, there is no adequate clinical evidence that metastasis of PALN is one of the absolute contraindications to pancreatic resection. PALN is removed routinely during operative procedure for pancreatic head cancer in our center and clinicopathological data of patients with pancreatic head cancer who received surgical resection in recent years are collected and analyzed with the aim of investigating the relationship between prognosis and PALN, and exploring prognostic factors in patients with and without metastasis of PALN, respectively.

## Materials and Methods

### Patients

Clinicopathological data of patients with pancreatic head cancer who underwent R0 or R1 pancreaticoduodenectomy between February 2008 and October 2015 were collected. Patients with R2 pancreatic resection and patients with neoadjuvant therapy were excluded from this study. Pancreatic head cancer was confirmed by intraoperative exploration and postoperative pathology. Patients with carcinomas of lower end of common bile duct, ampulla, duodenal papilla, and uncinate process, who also underwent pancreaticoduodenectomy, were not enrolled in this study. The protocol and procedures employed were reviewed and approved by the institutional review committee of our hospital.

### Preoperative preparations

Tumor markers, liver and kidney function, coagulation function, and blood routine were examined before operation, and contrast‐enhanced CT of abdomen, MRI of pancreas, and high‐resolution CT of lung were performed for all patients aimed at detecting distant metastasis and predicting resectability of pancreatic carcinoma. Contrast‐enhanced CT and MRI combined with angiography (CTA or MRA) were used for patients with suspected vascular invasion. Pulmonary function test and ultrasonic cardiogram were provided for patients with the age more than 60 years.

### Operation and adjuvant therapy

Standard Whipple procedures were carried out, and there were no patients received operation type of pylorus preserving pancreaticoduodenectomy. Extended lymphadenectomy was performed for all patients in this study. PALN which was defined as no. 16 was sampled by harvesting the lymphocellular aortocaval tissue from the upper part of the celiac trunk to the upper part of the origin of the inferior mesenteric artery. For patients with invasion of portal or superior mesenteric vein, pancreaticoduodenectomy combined with en bloc resection of PV/SMV was performed, and the choice of reconstruction types was determined by the different ranges of vascular invasion. Generally, partial venous excision with direct closure (venorrhaphy) by suture closure or using a patch was performed when the range of vascular invasion was less than one fourth of circumference of PV/SMV wall. Segment excision of PV/SMV should be performed if the range of vascular invasion was more than one fourth of circumference of the vessel wall. Reconstruction by using allogeneic vein was suggested if the length of PV/SMV removed was more than 2 cm. Postoperative adjuvant treatment of gemcitabine‐ or 5‐Fu‐based chemotherapy depending on physicians' choice and patients' condition was employed within 12 weeks after operation.

### Follow‐up

We followed up patients every 2 months with physical examination, laboratory tests, and image examinations. CT examination was performed semiannually and Doppler B ultrasound was performed bimonthly for patients. The follow‐up time was from 6 to 82 months, and the last follow‐up date was May 2016.

### Statistical analysis

SPSS 16.0 (IBM, Chicago, Illinois, USA) was used for data analysis. A *P *< 0.05 was considered to be statistically significant. Categorical variables were analyzed by chi‐square test, and continuous variables were analyzed by the Student's *t*‐test. Survival analysis was carried out using the Kaplan–Meier method with the log‐rank test. Cox's proportional hazards model was used to test for independent risk factors associated with survival.

## Results

### General conditions

A total of 233 patients with pancreatic head cancer who underwent surgical resection were enrolled in this study. The number of male and female patients was 108 and 125, respectively. The age of patients ranged from 40 to 79 years, with a median age of 57 years, and the CA19‐9 level before operation ranged from 17 to 1328 U/mL, with a median of 144 U/mL. Sixty‐nine patients underwent unintentional weight loss and 25 patients suffered from back pain preoperatively among these patients.

### Correlations between clinicopathological factors and survival in all patients

**Figure 1:**
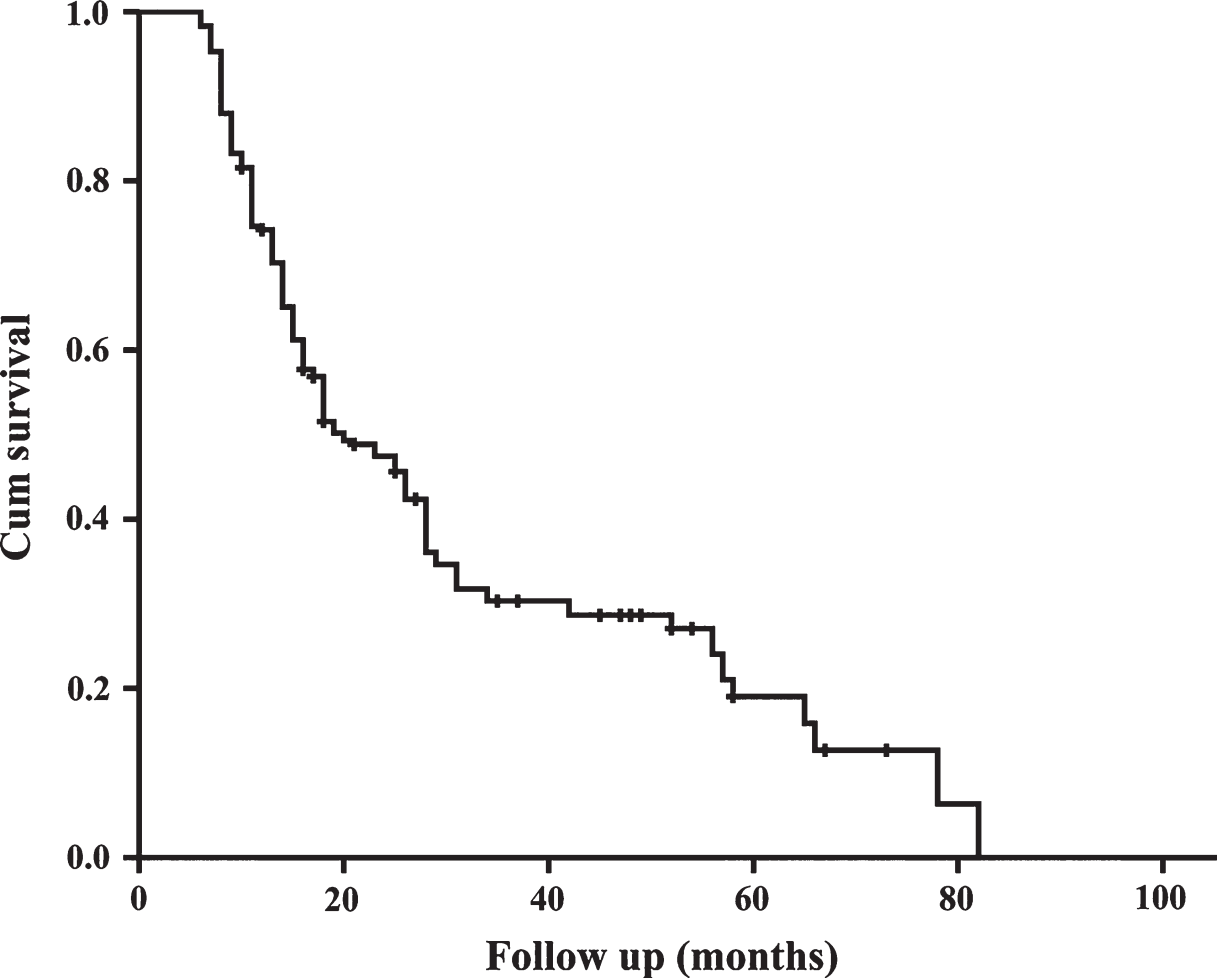
The overall survival curve of all 233 patients.

The 5‐year OS was 19.0% for all patients in this study (Fig. 1). As shown in Table 1, univariate analysis revealed that gender and age were not associated with 5‐year OS, while patients' conditions including preoperative CA19‐9 level, unintentional weight loss, and back pain had obvious influence on postoperative survival; pathological factors including tumor size, tumor differentiation, T status, N status, portal or superior mesenteric vein invasion, surgical margin status, number of lymph node retrieved, and the status of PALN were associated with survival. Patients with adjuvant therapy had significantly better prognosis compared with patients without. Multivariate analysis confirmed that preoperative CA19‐9 level, tumor size, T status, N status, and adjuvant therapy were independent prognostic factors (Table 2).

**Table 1:** Univariate analysis of prognostic factors for all patients with pancreatic head cancer

	No. of patients (*n* = 233)	5‐year OS (%)	*P*‐value
Gender			
Male	108	19.0	0.498
Female	125	18.7	
Age, years			
<60	141	16.9	0.186
≥60	92	23.5	
ASA			
2	110	17.9	0.969
3	123	24.5	
Preoperative CA19‐9 level, U/mL			
<100	124	29.7	<0.001
≥100	109	5.8	
Unintentional weight loss			
Yes	69	7.0	<0.001
No	164	22.8	
Back pain			
Yes	25	0	<0.001
No	208	21.2	
Adjuvant chemotherapy			
Yes	188	25.0	<0.001
No	45	2.2	
Tumor size, cm			
<2	44	50.8	<0.001
≥2	189	10.4	
Tumor differentiation			
Poorly	147	14.0	<0.001
Moderately/well	86	26.3	
T status			
T_1–2_	116	40.2	<0.001
T_3–4_	117	0	
N status			
Negative	72	42.7	<0.001
Positive	161	7.8	
Portal or superior mesenteric vein invasion			
Yes	38	0	<0.001
No	195	22.9	
Margin involvement			
Yes	37	3.2	0.034
No	196	23.0	
Number of lymph node retrieved			
<15	70	13.0	0.001
≥15	163	23.0	
Para‐aortic lymph node metastasis			
Yes	44	0	<0.001
No	189	22.9	

ASA, American Society of Anesthesiologists; OS, overall survival.

**Table 2:** Multivariate analysis of prognostic factors for all of the 233 patients with pancreatic head cancer

	5‐year OS		
	RR	CI 95%	*P*‐value
Preoperative CA19‐9	1.660	1.123–2.452	0.011
Unintentional weight loss	0.861	0.553–1.341	0.509
Back pain	1.516	0.869–2.644	0.143
Adjuvant chemotherapy	0.668	0.456–0.978	0.038
Tumor size	2.077	1.244–3.469	0.005
Tumor differentiation	0.874	0.595–1.284	0.492
T status	2.714	1.753–4.202	<0.001
N status	2.786	1.799–4.315	<0.001
Margin involvement	0.886	0.592–1.326	0.556
Portal or superior mesenteric vein invasion	0.757	0.471–1.217	0.251
Lymph node retrieved	0.951	0.674–1.341	0.773
Para‐aortic lymph node metastasis	0.944	0.558–1.597	0.830

OS, overall survival.

### Correlations between clinicopathological factors and survival according to the status of PALN

**Figure 2:**
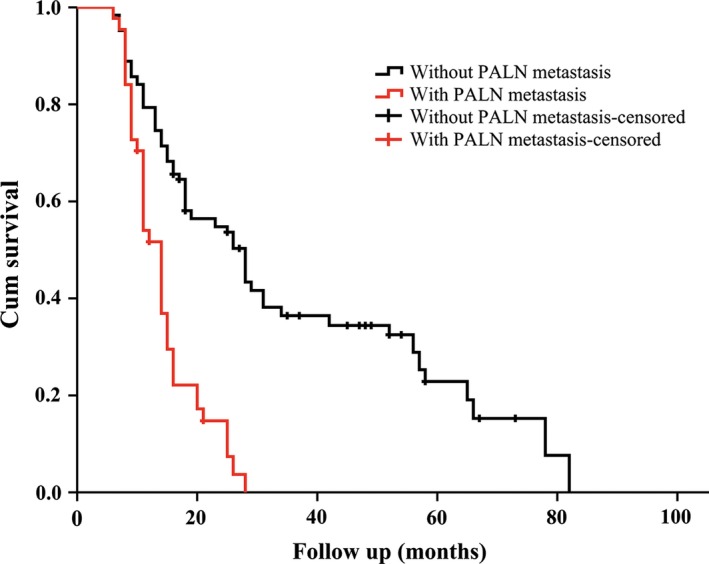
The respective survival curves of 44 patients with metastasis of para‐aortic lymph node (PALN) and 189 patients without metastasis of PALN.

Of the 233 patients, metastasis of PALN was found in 44 patients, the positive rate of PALN metastasis was 18.9% in this study. Number of PALN removed ranged from one to four for all patients and the number of PALN in which cancer cells were detected ranged from one to three in patients with metastasis of PALN in this study. There was statistically significant difference according to OS between patients with and without metastasis of PALN. The 1‐, 2‐, 3‐, and 5‐year OS rates in patients without metastasis of PALN were 79.4%, 54.8%, 36.4%, and 22.9%, respectively. While the 1‐, 2‐, and 3‐year survival rates were 54.0%, 14.8%, and 0%, respectively, in patients with metastasis of PALN (Fig. 2). Univariate analysis showed that preoperative CA19‐9 level, unintentional weight loss, back pain, tumor size, T status, N status, surgical margin status, porta or superior mesenteric vein invasion, number of lymph node retrieved, and adjuvant therapy were associated with 5‐year OS in patients without metastasis of PALN (Table 3). Multivariate analysis revealed that back pain, tumor size, T status, N status, portal or superior mesenteric vein invasion, and adjuvant therapy were independent prognostic factors in patients without PALN metastasis (Table 4). Considering that no survival patients at 3 years, we analyzed the relationship between clinicopathological factors and 2‐year OS for patients with metastasis of PALN. As shown in Table 3, the only one influence factor for 2‐year OS was adjuvant therapy in patients with metastasis of PALN. One point needed attention was that there was no correlation between number of positive PALN and 2‐year OS.

**Table 3:** Univariate analysis of prognostic factors for patients with and without metastasis of PALN

	16 (−)			16 (+)		
	No.	5‐year OS (%)	*P*‐value	No.	2‐year OS (%)	*P*‐value
Gender						
Male	87	23.2	0.636	21	0	0.150
Female	102	22.3		23	26.1	
Age, years						
<60	117	20.5	0.167	24	12.5	0.657
≥60	72	27.9		20	17.9	
ASA						
2	84	22.3	0.608	26	21.0	0.998
3	105	28.5		18	6.1	
Preoperative CA19‐9 level, U/mL						
<100	113	31.5	<0.001	11	41.6	0.062
≥100	76	8.2		33	6.3	
Unintentional weight loss						
Yes	42	10.3	<0.001	27	12.7	0.776
No	147	25.3		17	16.0	
Back pain						
Yes	6	0	<0.001	19	5.8	0.158
No	183	23.6		25	21.4	
Adjuvant chemotherapy						
Yes	153	29.8	<0.001	36	18.7	0.001
No	36	5.6		9	0	
Tumor size, cm						
<2	39	57.3	<0.001	5	0	0.141
≥2	150	12.7		39	16.7	
T status						
T_1–2_	114	40.9	<0.001	2	0	0.617
T_3–4_	95	0		32	15.5	
N status						
Negative	72	43.3	<0.001	–	–	–
Positive	117	10.1		–	–	
Margin involvement						
Yes	26	3.8	0.036	11	0	0.349
No	163	27.3		33	20.2	
Portal or superior mesenteric vein invasion						
Yes	15	0	0.018	23	9.3	0.160
No	174	25		21	20.6	
Lymph node retrieved						
<15	56	16.1	0.001	14	16.3	0.961
≥15	133	27.3		30	14.1	
Number of positive PALN						
1	–	–	–	27	16.8	0.428
>1	–	–		17	11.8	

OS, overall survival; PALN, para‐aortic lymph node.

**Table 4:** Multivariate analysis of prognostic factors for the 189 patients without metastasis of PALN

	5‐year OS in patients with 16 (−)		
	RR	CI 95%	*P*‐value
Preoperative CA19‐9	1.565	0.994–2.464	0.053
Unintentional weight loss	1.305	0.642–1.669	0.887
Back pain	3.720	1.432–9.662	0.007
Adjuvant chemotherapy	0.607	0.289–0.945	0.027
Tumor size	2.718	1.532–4.824	0.001
T status	2.771	1.730–4.437	<0.001
N status	2.950	1.887–4.610	<0.001
Margin involvement	0.886	0.592–1.326	0.556
Portal or superior mesenteric vein invasion	0.412	0.212–0.801	0.009
Lymph node retrieved	0.777	0.515–1.171	0.227

OS, overall survival; PALN, para‐aortic lymph node.

## Discussion

Although there is some evidence that the outcome is improving over time, prognosis of pancreatic cancer remains extremely poor and surgical indication has not been greatly changed 2. Several factors are associated with prognosis of pancreatic cancer, such as tumor size, depth of invasion, lymph node status, and surgical margin status 6, 7. PALN has been classified as nonregional lymph nodes, and metastasis of PALN has been recognized as distant metastasis. A limited number of studies about PALN had been carried out, and the metastatic rate of PALN ranged from 11% to 26% according to these studies 7, 12, 20. In this study, the lymph node involvement was 69.1%, and the metastatic rate of PALN was 18.9%, which was within the range reported previously.

Extremely poor prognosis in pancreatic cancer patients with metastasis of PALN had been confirmed by several studies 20, 21, 22. As an example, some previous studies reported that the median survival time of patients with PALN involvement was only between 5.1 and 15.7 months 4, 7, 20, 23, and some others reported that 2‐year survival rates ranged from 0% to 18% if metastasis to PALN in patients with pancreatic cancer 21, 22, 24. There are exceptions, of course. Patients with involvement of PALN had relatively better prognosis in a study designed by Sho et al. 12 compared to others; The 1‐, 2‐, 3‐, and 5‐year survival rates for patients with PALN metastasis were 63.8%, 30.0%, 16.7%, and 6.8%, respectively, although they were significantly lower than patients without metastasis of PALN. In our study, the 1‐, 2‐, and 3‐year survival rates were 54.0%, 14.8%, and 0% in patients with metastasis of PALN, but the 1‐, 2‐, 3‐, and 5‐year survival rates were 79.4%, 54.8%, 36.4%, and 22.9% in patients without metastasis of PALN. Our results were similar to that reported by Murakami et al. 7, the 1‐, 2‐, 3‐, and 5‐year OS rates were 79%, 49%, 29%, and 23% in patients without metastasis of PALN, whereas 1‐, 2‐, and 3‐year OS rates were 53%, 12%, and 0% in patients with metastasis of PALN in his study.

In the study designed by Murakami et al. 7, univariate analysis showed that metastasis of PALN was one of the prognostic factors, while multivariate survival analysis revealed that it was not an independent prognostic factor, similar results were found in study designed by Sho et al. 12, whereas Doi et al. 4 reported that metastasis to PALN was the single independent factor associated with a shorter survival time. Our study also showed that the status of PALN was significantly associated with prognosis for all patients, but it was not one independent prognostic factor confirmed by multivariate survival analysis. For patients without metastasis of PALN, preoperative CA19‐9 level, unintentional weight loss, back pain, adjuvant chemotherapy, tumor size, T status, N status, margin involvement, portal or superior mesenteric vein invasion, and lymph node retrieved were associated with survival in our study, and back pain, tumor size, T status, N status, and portal or superior mesenteric vein invasion were independent prognostic factors. However, only adjuvant therapy was associated with survival for patients with metastasis of PALN. Application of adjuvant systemic chemotherapy was crucial in PALN‐positive patients based on our results, although reports concerning the survival benefits of postoperative adjuvant chemotherapy for patients with metastasis of PALN were scarce. Selection of chemotherapy regimen was dependent on several factors including physicians' suggestion, ECOG of patients, morbidity profile, age, and so on. Either FOLFIRINOX (leucovorin, fluorouracil, irinotecan, and oxaliplatin) or gemcitabine plus nanoparticle albumin‐bound (NAB) paclitaxel can be used for patients as first‐line regimen. Adjuvant chemotherapy is typically initiated within 6–12 weeks of surgical resection, there are a few of studies about the influence of interval on prognosis of pancreatic cancer. For instance, a new study designed by Mirkin et al. 25 suggested that time to the initiation of adjuvant chemotherapy does not impact survival in patients with resected pancreatic cancer.

In conclusion, pancreatic head cancer with metastasis of PALN had poor prognosis compared with that without metastasis of PALN, but status of PALN was not the independent prognostic factor. Patients with or without metastasis of PALN had different prognostic factors, several factors had impact on survival for patients without metastasis of PALN, whereas adjuvant therapy was the only prognostic factor for patients with metastasis of PALN.

## Conflict of Interest

There are no potential conflicts of interest for each of the authors.
